# Long-term follow-up of passive containment surgery in patients with aortic regurgitation

**DOI:** 10.1093/icvts/ivab273

**Published:** 2021-10-19

**Authors:** Fredrik Bredin, Anders Franco-Cereceda

**Affiliations:** Section of Cardiothoracic Surgery, Department of Molecular Medicine and Surgery, Karolinska Institute, Stockholm, Sweden

**Keywords:** Aortic valve replacement, Aortic regurgitation, Ventricular remodelling, Passive containment surgery

## Abstract

The goal of this case-control study was to evaluate the long-term effects on cardiac dimensions, cardiac function and coronary circulation in patients with aortic regurgitation (AR) and left ventricular dilatation undergoing aortic valve replacement and application of the Acorn CorCap cardiac support device Of 10 patients with AR and ventricular dilatation who had a mechanical aortic valve implanted, 5 in addition received the cardiac support device. Cardiac dimensions and cardiac function were measured by echocardiography preoperatively and 1, 5 and 10 years postoperatively. The coronary circulation was assessed by computed tomography angiography. After aortic valve replacement, there was a rapid and sustained decrease in cardiac dimensions. This result did not differ after application of the cardiac support device. Improvement in cardiac function remained unchanged in both groups at the 10-year follow-up. None of the patients had developed any signs of coronary artery disease. Application of the Acorn CorCap cardiac support device in patients with AR and left ventricular dilatation did not add to the reversed remodelling or cardiac function at the long-term follow-up compared to aortic valve replacement alone.

## INTRODUCTION

Aortic valve replacement (AVR) is indicated in the absolute majority of patients with symptomatic aortic regurgitation (AR). Patients with AR and left ventricular (LV) dysfunction or severe LV dilatation should be evaluated for AVR even if they are asymptomatic [1].

Heart failure is an increasing problem in the ageing Western population. Despite improvements in treatment, the prognosis for severe heart failure is still poor [2]. The failing heart undergoes numerous structural and functional changes referred to as ventricular remodelling [3].

The Acorn CorCap cardiac support device (CSD) is a mesh-like polyester fabric with bidirectional compliance. It was developed primarily to be positioned around the failing heart in order to reduce wall stress and reshape the heart from a spherical shape to an ellipsoidal shape, thus facilitating a reversed remodelling of the heart [4]. Numerous animal studies in different heart failure models indicated reversal of molecular and cellular abnormalities after CSD application. The intermediate time results of CSD application with or without concomitant mitral valve surgery have previously been evaluated [5, 6].

We earlier presented the short-term effects of using the CSD in patients with AR and LV dilatation [7]. The long-term effects on cardiac function and coronary circulation following application of the CSD are unknown. The goal of the present case–control study was to evaluate the long-term effects of CSD application combined with AVR in patients with AR and LV dilatation by echocardiographic measurements of cardiac dimensions and cardiac function and to evaluate the presence of coronary artery disease (CAD) by computed tomography angiography (CTA).

## PATIENTS AND METHODS

Between April 2003 and March 2006, a total of 10 male patients with AR graded as 3–4/4, ventricular dilatation with a left ventricular end diastolic diameter (LVEDD) >70 mm, no other valvular disorder and normal results on coronary angiograms were accepted for AVR with a mechanical prosthesis. The first 5 patients agreed to participate in a pilot study evaluating the effects of the concomitant application of CSD in addition to a mechanical valve prosthesis. Five consecutive patients undergoing AVR with a mechanical prothesis as the sole procedure served as controls. The study was approved by the local ethics committee at the Karolinska University Hospital, the choice of valve prothesis was discussed preoperatively and written consent was obtained from all patients.

The preoperative characteristics of the patients are presented in Table 1.

**Table 1:** Pre- and perioperative patient characteristics

Patient number	Age (years)	Native valve	Prothesis size (mm)	LVEF (%)	LVEDD (mm)
1	45	Bicuspid	29	50	75
2	44	Bicuspid	25	40	75
3	61	Tricuspid	29	50	73
4	38	Bicuspid	25	40	74
5	35	Tricuspid	25	30	68
6	36	Bicuspid	27	45	75
7	57	Tricuspid	23	50	70
8	62	Tricuspid	25	30	70
9	69	Bicuspid	25	55	78
10	43	Bicuspid	27	55	73

LVEF: left ventricular ejection fraction; LVEDD: left ventricular end diastolic diameter.

Prior to surgery and 1, 5 and 10 years postoperatively all patients were evaluated by transthoracic echocardiography. All examinations were performed in an unblinded manner. The left ventricular dimension was measured as LVEDD, and the ejection fraction was calculated using the modified Simpsons rule.

All patients but 1 (due to attenuated kidney function) in the CSD group underwent a CTA examination of their coronary arteries 8–10 years postoperatively. CTA is a well-validated cardiac imaging method used to assess the presence or absence of CAD [8]. The examinations were performed to detect CAD using dual source 2 × 64-row multidetector computed tomography (Siemens Somatom Definition Flash; Siemens Healthcare, Forchheim, Germany). All examinations were analysed by the same thoracic radiologist, who was blinded to all clinical information.

### Statistical analyses

Data are presented as mean ± standard deviation. The Mann–Whitney *t*-test and the repeated measures analysis of variance were used to evaluate possible statistical changes between the groups and interactions in the groups over time.

## RESULTS

All patients survived the surgical procedure and could leave the hospital. Postoperative follow-up 1, 5 and 10 years postoperatively is complete. Data are given as mean and standard deviation (SD).

### Cardiac dimensions

Echocardiographic examinations showed a sustained reduction in cardiac dimensions measured as the LVEDD. The LVEDD decreased from 74 (SD 1.3) mm and 72 (SD 3.8) mm to 58 (SD 0.4) mm and 54 (SD 7.5) mm at the 10-year postoperative follow-up in patients subjected to AVR combined with the application of the CSD and controls, respectively (Table 2).

**Table 2:** The left ventricular end diastolic diameter and left ventricular ejection fraction measurements during the study period in patients subjected to aortic valve replacement and the cardiac support device or to aortic valve replacement as the sole procedure

	AVR + CSD	AVR
LVEF (%)		
Preoperatively	49 (SD : 3.7)	51 (SD : 4.7)
1 year postoperatively	50 (SD : 8.8)	55 (SD : 7.2)
5 years postoperatively	56 (SD : 6.5)	54 (SD : 2.5)
10 years postoperatively	56 (SD : 4.2)	55 (SD : 3.5)
LVEDD (mm)		
Preoperatively	74 (SD : 1.3)	72 (SD : 3.8)
1 year postoperatively	54 (SD : 5.6)	53 (SD : 8.9)
5 years postoperatively	55 (SD : 7.2)	50 (SD : 4.2)
10 years postoperatively	58 (SD : 4.4)	54 (SD : 7.5)

*P* > 0.05 between groups at all times.

AVR: aortic valve replacement; CSD: cardiac support device; LVEDD: left ventricular end diastolic diameter; LVEF: left ventricular ejection fraction; SD: standard deviation.

### Cardiac function

There were no changes in cardiac function as measured by the LVEF in either of the 2 groups of patients who were operated on (Table 2).

### Angiographic examination

A total of 57 and 60 segments were evaluated in the AVR + CSD and AVR groups, respectively. None of the patients had developed any significant coronary artery disease.

## DISCUSSION

Pharmacological therapies and different surgical procedures have been shown to induce favourable changes in myocyte and myocardial functions as well to reduce cardiac dimensions. The term reversed remodelling has been used to describe the mechanics leading to improvements in clinical manifestations and in the prognosis for heart failure patients.

In patients with AR undergoing AVR, there is a correlation between the early reduction of LV dimensions and improvement of LV function. Furthermore, a lack of these signs of reversed remodelling is associated with poor prognosis [9]. The goal of this study was to investigate the potential beneficial long-time effects of the CSD combined with AVR in patients with AR and LV dilatation on cardiac dimensions, cardiac function and coronary circulation. Our findings demonstrated no differences in changes regarding LV function or LV dimensions between patients undergoing the CSD in addition to AVR and control patients undergoing AVR as the sole procedure. No negative effects on coronary circulation or development of CAD could be diagnosed at the long-term follow-up with CTA. Although the use of the CSD has almost vanished due to difficulties in identifying selection criteria for applying CSD, the absence of significant CAD is of importance because numerous patients worldwide have received the CSD.

### Limitations

A limitation of this study is the small number of patients included because of the limited number of patients in our institution meeting the inclusion criteria.

A much larger number of patients would be needed for a well-powered evaluation. However, any major significant effects on the studied parameters should have been detected in the present study population during the study time.

## CONCLUSION 

In conclusion, this case–control study has shown that the application of the CSD concomitant with AVR in patients with AR and LV dilatation at the long-term follow-up does not affect LV dimension or LV function compared to AVR alone and that the CSD adds no beneficial effects for these patients. No significant effect on coronary artery morphology was seen in patients receiving the CSD; however, future acquired coronary stenosis not amenable to percutaneous interventions may pose a risk for patients subjected to CSD application. Technical challenges in patients undergoing redo open heart surgery following application of the CSD have been reported previously [10].
